# Resource use, costs, and approval times for planning and preparing a randomized clinical trial before and after the implementation of the new Swiss human research legislation

**DOI:** 10.1371/journal.pone.0210669

**Published:** 2019-01-11

**Authors:** Benjamin Speich, Nadine Schur, Dmitry Gryaznov, Belinda von Niederhäusern, Lars G. Hemkens, Stefan Schandelmaier, Alain Amstutz, Benjamin Kasenda, Christiane Pauli-Magnus, Elena Ojeda-Ruiz, Yuki Tomonaga, Kimberly McCord, Alain Nordmann, Erik von Elm, Matthias Briel, Matthias Schwenkglenks, a collaboration of the MARTA (MAking Randomized Trials Affordable) and ASPIRE (Adherence to Standard Protocol Items: REcommendations for interventional trials) Study Groups

**Affiliations:** 1 Basel Institute for Clinical Epidemiology and Biostatistics, Department of Clinical Research, University of Basel and University Hospital Basel, Basel, Switzerland; 2 Institute of Pharmaceutical Medicine, University of Basel, Basel, Switzerland; 3 Clinical Trial Unit, Department of Clinical Research, University of Basel and University Hospital Basel, Basel, Switzerland; 4 Department of Health Research Methods, Evidence, and Impact, McMaster University, Hamilton, ON, Canada; 5 Swiss Tropical and Public Health Institute, and Division of Infectious Diseases and Hospital Epidemiology, University of Basel and University Hospital Basel, Basel, Switzerland; 6 Department of Medical Oncology, University of Basel and University Hospital Basel, Basel, Switzerland; 7 National Centre for Epidemiology, Instituto de Salud Carlos III, Madrid, Spain; 8 Epidemiology, Biostatistics and Prevention Institute, University of Zurich, Zurich, Switzerland; 9 Cochrane Switzerland, Institute of Social and Preventive Medicine (IUMSP), Lausanne University Hospital, Lausanne, Switzerland; Federico II University of Naples, ITALY

## Abstract

**Background:**

The preparation of a randomized controlled trial (RCT) requires substantial resources and the administrative processes can be burdensome. To facilitate the conduct of RCTs it is important to better understand cost drivers. In January 2014 the enactment of the new Swiss Legislation on Human Research (LHR) considerably changed the regulatory framework in Switzerland. We assess if the new LHR was associated with change in (i) resource use and costs to prepare an RCT, and (ii) approval times with research ethics committees (RECs) and the regulatory authority Swissmedic.

**Methods:**

We surveyed investigators of RCTs which were approved by RECs in 2012 or in 2016 and asked for RCT preparation costs using a pre-specified item list. Additionally, we collected approval times from RECs and Swissmedic.

**Results:**

The response rates of the investigator survey were 8.3% (19/228) for 2012 and 16.5% (47/285) in 2016. The median preparation cost of an RCT was USD 72,400 (interquartile range [IQR]: USD 59,500–87,700; n = 18) in 2012 and USD 72,600 (IQR: USD 42,800–169,600; n = 35) in 2016. For single centre RCTs a median REC approval time of 82 (IQR: 49–107; n = 38) days in 2012 and 92 (IQR: 65–131; n = 63) days in 2016 was observed. The median Swissmedic approval time for any clinical trial was 27 (IQR: 19–51; n = 213) days in 2012 and 49 (IQR: 36–67; n = 179) days in 2016. The total duration for achieving RCT approval from both authorities (REC and Swissmedic) in the parallel submission procedure applied in 2016 could not be assessed.

**Conclusion:**

Based on limited data the costs to plan and prepare RCTs in Switzerland were approximately USD 72,000 in 2012 and 2016. For effective and valid research on costs and approval times of RCTs a greater willingness to share cost information among investigators and more collaboration between stakeholders with data linkage is necessary.

## Introduction

Randomized controlled trials (RCTs) provide the most reliable evidence on therapeutic and preventive interventions for decision-making in clinical practice and health policy [1]. Although the available evidence on RCT costs is limited, it is assumed that the administrative burden as well as the costs for RCTs have increased substantially over the last decades [1–4]. This is problematic for two reasons: First, pharmaceutical companies argue that high prices for new interventions are justified by the high development costs which are mostly driven by costs for RCTs [5–7]. Second, especially for academic investigators it becomes increasingly difficult to conduct clinical trials [2, 8]. For example, the number of trials performed dropped drastically after the introduction of The European Union Clinical Trials Directive in 2004 [8]. When academic clinical trials are becoming unfeasible, many important clinical questions without prospect for financial profit will remain unanswered. To improve this situation, it is important to know what causes the burden to conduct clinical research (i.e. what are cost drivers that hamper the planning and conduct of RCTs). However, a systematic review showed that transparently available cost data for RCTs are sparse and the usefulness of the available data is limited [9].

In January 2014, the regulatory framework for clinical research in Switzerland considerably changed with the enactment of the new law on research with human beings (Human Research Act, HRA) and its ordinances (Swiss Legislation on Human Research, LHR) [10]. The new LHR was implemented to protect the dignity, privacy and health of human beings involved in research, but should also help to ensure the quality of research and improve transparency as well as to create favourable conditions for researchers [10, 11]. There is a notion that with the introduction of the LHR, additional administrative expenses may have occurred for some types or parts of clinical studies (e.g. study registration, liability insurance, or clarification of study risk category). At the same time, simplifications may have been achieved by introducing the parallel submission process to research ethics committees (RECs) and the regulatory authority Swissmedic (i.e. Swiss regulatory authority for drugs and medical devices) as well as a lead REC process which should reduce approval time for new study protocols and alleviates inter-regional differences between RECs [10]. Furthermore, the newly introduced risk categorisation allows for the differentiation of low (category A) and mid- to high-risk (categories B & C) trials (e.g. categorisation for RCTs with pharmaceutical treatments is linked to the approval status of the studied treatment), with significant facilitations for the conduct of category A studies that are particularly prevalent at academic institutions [12]. Before the introduction of the LHR, RECs had 30 days from receipt of a study protocol to decide whether the study should be approved, revised or rejected. Swissmedic also had 30 days to request revisions, and both authorities were able to restart the deadline if revisions were necessary. Since the implementation of the new LHR, both institutions have, in parallel in case of simultaneous submission, a first phase of seven days to check if all formal requirements are met, and then an additional 30 days to decide if a study can be approved or if amendments are required. In some cases this deadline can be expanded (i.e. additional 15 days for RECs for multicentre studies; 30 days for Swissmedic if a drug is used for the first time in humans or manufactured in a new process) [13, 14].

In the present study, we aimed to assess the costs to plan and prepare RCTs in Switzerland as well as approval times with RECs and Swissmedic. We further aimed to investigate whether the new LHR was associated with any change in resource use and costs or in approval times. Hence, we compared data from RCTs approved by Swiss RECs in 2012 (two years before the introduction of the LHR) with RCTs approved in 2016 (two years after the introduction of the LHR).

## Materials and methods

This study is reported according to the Strengthening the Reporting of Observational Studies in Epidemiology (STROBE, http://www.equator-network.org/reporting-guidelines/strobe/) statement, as described in the supporting file (S1 File).

### Study sample

For the present study we used RCT protocols that were included in the ongoing Adherence to Standard Protocol Items: Recommendations for Interventional Trials (ASPIRE) project. ASPIRE evaluates the reporting quality of study protocols for RCTs according to the Standard Protocol Items: Recommendations for Interventional Trials (SPIRIT) statement [15, 16]. We included RCT protocols that were approved by a Swiss REC in 2012 (before the implementation of the new LHR) or 2016 (after the implementation of the new LHR). RCTs were defined as prospective studies in which patients, or groups of patients, were randomly assigned to different interventions to evaluate effects on health outcomes. We excluded studies with healthy volunteers, or primarily assessing health economic issues, as well as pilot studies, studies with pseudo-randomisation, animal studies, and tissue bank studies. For each eligible RCT we recorded characteristics including sponsor, funding source, number of patients, number of study centres, number of involved countries, site of study initiation (Switzerland vs. other), if a clinical trial unit (CTU) or contract research organisation (CRO) was involved in the RCT, and the LHR risk category (applicable only for study protocols approved in 2016).

### Estimation of resource use and costs of RCT preparation

In a previous study we had developed a comprehensive cost item list for RCTs that was further refined in a case study on the resource use and costs of two investigator-initiated RCTs [17]. This list was sent to all principal investigators of the RCTs included in the ASPIRE project (approved RCT protocols in 2012 or 2016), as a collection tool on resource use and costs (see S2 File). We informed them by letter about the purpose of the cost data collection and asked them about resource use and costs during the preparation phase (conception, planning, and preparation) of their respective RCT. In the first part of the item list, working time efforts for all personnel had to be estimated for specific items (e.g. development of grant proposals, writing study protocol, communication with study sites). Investigators were also asked about salary levels for different staff categories (this part was labelled as “optional”). On this basis, working time estimates were translated into cost data. In the second part of the item list, investigators were asked about fixed costs (e.g. REC fees, insurance costs, fixed costs for any kind of support). All received cost item lists filled in by investigators were checked for completeness. If forms were incomplete (i.e. contained missing data which could potentially be provided by the investigators; e.g. missing fixed costs), we contacted the investigators again and asked whether the missing data could be provided. As the part on salary levels was optional, missing information on salaries was imputed using median salaries per staff category (e.g. coordinating principal investigator, investigator, research nurse, medical staff (senior), medical staff (junior), and information technology support).

### Data on approval times

REC and Swissmedic, approval time were collected directly from these institutions. In addition, Interpharma (the association of pharmaceutical companies with research activities in Switzerland) provided us with aggregated REC and Swissmedic approval time data collected by pharmaceutical companies. At the level of RECs, for RCTs approved in 2012, we extracted the dates when the protocols were submitted to a Swiss REC, when the RECs gave the first response to the investigator, and when REC approved the RCT. For 2016, this information was directly available from the newly introduced Business Administration System for Ethics Committees (BASEC). In 2016, all approval times for multicentre RCTs were from lead-RECs (responsible for all issues relating to the trial; non-lead RECs check local conditions) [10]; while in 2012 the differentiation between lead- and non-lead RECs remained mostly unclear. We also requested from Swissmedic approval time data for all RCTs that required Swissmedic approval in 2012 and 2016. We aimed at matching Swissmedic and REC approval time data at the RCT level, on the basis of a joint identifier. It was planned, for each RCT which required approval from both authorities, to identify the date of the first submission (either to a Swiss REC or to Swissmedic) and the date when both competent authorities approved the RCT, as a basis for the calculation and descriptive statistical analysis of joint Swissmedic and REC approval time. Finally, we received data from Interpharma on approval times of RCT protocols at Swiss RECs and Swissmedic between 2012 and 2016. Interpharma collected these data through a survey of Swiss pharmaceutical companies that could enter approval time data for current clinical studies on a voluntary basis.

### Analysis

In terms of reported effort for the preparation phase of RCTs, we summarised total working time and costs (median, mean, IQR, minimum and maximum) for the RCTs approved in 2012 and 2016. All cost data were converted from Swiss Francs into USD (1 Swiss Franc = 1.018 USD; exchange rate 1. October 2018). We only included complete survey responses, except in an analysis of item specific costs. For each applicable item of the cost item list, we calculated the total working time effort per RCT (considering all individuals contributing to the same item). To account for non-included time, unforeseen tasks (e.g. walking between patients or buildings, discussing study) and for unproductive phases, we increased the calculated working time by 30% (“non-pre-specified time”) [18]. Salaries were increased by 20% to account for overhead costs. Descriptive subgroup analyses were conducted: (i) by type of sponsor (i.e. industry sponsor vs. non-industry sponsor); (ii) for single centre vs. multicentre RCTs; (iii) for RCTs with or without CTU/CRO involvement; and (iv) by risk category (only available for RCTs approved in 2016).

In sensitivity analyses, we calculated (i) results without adding 30% of non-pre-specified time; (ii) results without adding 30% of non-pre-specified time and without adding 20% overhead to salaries; and (iii) excluded all studies where we needed to impute the salaries of the study personnel. Finally, we descriptively summarised item specific costs for the preparation phase. For this analysis, we also included incomplete data sets whenever they reported time efforts or costs for a given item.

In the main analyses of REC and Swissmedic approval times, we considered the entire time period from submission to final approval. We also intended to calculate approval times corrected for (disregarding) times which the sponsor required to respond to requests from the authorities; finally, this was only possible for the data collected from Swissmedic). The different target timelines applying to REC and Swissmedic submissions were taken into account in the interpretation of the results [13, 14].

For the approval times collected directly from RECs, we present, for 2012 and 2016, mean, median, 25th to 75th percentile range (IQR), minimum and maximum for the following durations: (i) time from submission until approval by the REC; (ii) time from submission until first response by the REC; and (iii) time from first response until date of approval by the REC. Additionally, we conducted the same descriptive subgroup analyses as for the cost analyses. For RCTs approved in 2012, the available data did not allow us to distinguish lead RECs from non-lead RECs for multicentre RCTs, preventing a meaningful comparison of overall REC approval times in 2012 with overall REC approval times in 2016. However, for the subsample of single-centre RCTs a comparison was sensible, and so we used a two-sided Wilcoxon rank-sum test for unpaired samples to compare times from application to approval for single-centre RCTs in 2012 and 2016.

Swissmedic provided us with the dates of receiving a dossier, of acknowledgment of receipt, and of the Swissmedic decision, for clinical trials in general, i.e. not specifically for RCTs. Non-RCTs could not be identified and separated. We calculated the time from submission until approval and provide the same descriptive statistics as stated above. Subgroup analyses by type of sponsor according to the Swissmedic classification (i.e. industry trials vs. investigator initiated trials (IIT) vs. trials conducted by collaborative study groups) were performed. We excluded trials which had to be submitted to the Federal Office of Public Health for further evaluation, as we did not have information on when these were approved. The Swissmedic data did not include an identification code that would have enabled us to match Swissmedic and REC approval time data at the RCT level. In consequence, we had no means of assessing combined Swissmedic and REC approval times.

Data from Interpharma are presented as received.

## Results

**Fig 1:**
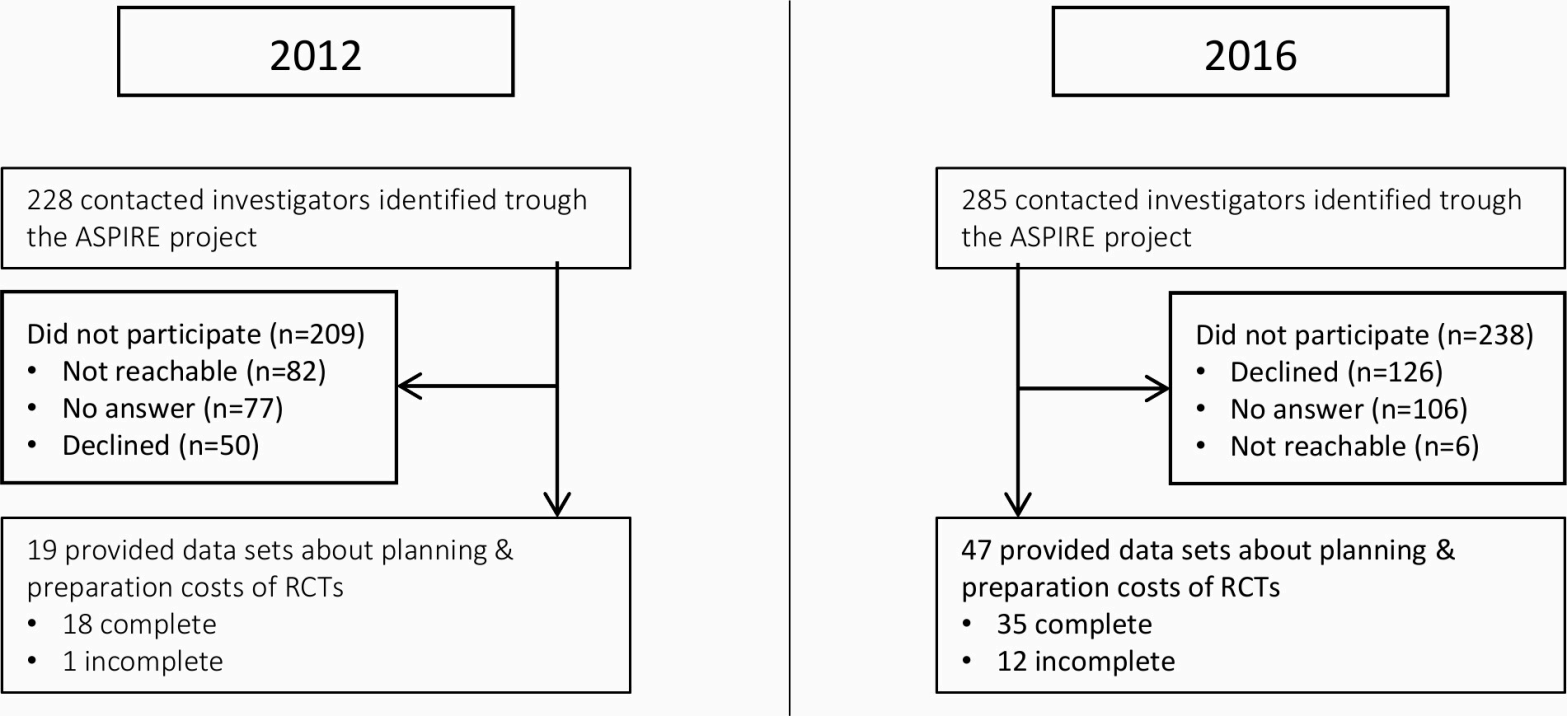
Flow chart summarising the collection of RCT preparation costs. Abbreviations: RCT, randomized clinical trial; ASPIRE, Adherence to SPIrit statement REcommendations.

We contacted and asked investigators of a total of 228 and 285 RCTs approved in 2012 and 2016, respectively, to provide us with data on resource use and costs for the preparation phase of their RCTs (Fig 1). Out of the sample of RCTs initiated before the implementation of the LHR (2012), we received preparation and planning costs for 19 RCTs (response rate: 8.3%) that were incomplete in one case (here, all estimates of working time effort were missing). Out of the RCTs that were approved in 2016 (after implementation of the LHR), we received a total of 47 data sets of preparation and planning costs (response rate: 16.5%). Twelve of these were incomplete and could only be used for the evaluation of costs of specific items.

In 2012, nine of the 18 RCTs with complete datasets were multicentre RCTs with a median of three centres (IQR: 2–3 centres; max: 70 centres). None of these RCTs was international. Two RCTs were initiated by industry sponsors and 16 by non-industry sponsors. (Table 1). For 2016, we received complete datasets for nine multicentre (median of 3 centres; IQR: 2–6 centres; max: 7 centres; including 2 international RCTs) and 26 single centre RCTs. Four RCTs were initiated by industry sponsors and 31 by non-industry sponsors. In 2012, the preparation phase of RCTs required, in the median, 113 days (IQR: 51–190 days) in 2012 and 133 working days (IQR: 79–239 days) in 2016 (Table 1). The median costs to plan and prepare an RCT were around USD 72,000 in 2012 as well as in 2016. Results stratified by study characteristics (i.e. single centre, multicentre, industry, non-industry, CTU involvement, no CTU-involvement and different risk categories) are presented in Table 1. Median costs remained relatively stable across strata, except in the case of multicentre RCTs in 2016 (USD 141,000 based on nine RCTs). Results from the sensitivity analyses are presented in Table 2. Excluding studies where we needed to impute salaries (Table 2, scenario 3) led to a substantially decreased cost estimate for 2016 (USD 56,000 based on 14 RCTs). Costs per item were, in general, comparable between 2012 and 2016 (Table 3). It is interesting to note that, while protocol and case report form development contributed most to the RCT preparation costs (i.e. median of USD 34,774 in 2012 and USD 39,503 in 2016), other items of less obvious relevance also resulted in considerable costs (e.g. communication [median of USD 7,322 in 2012 and USD 6,506 in 2016], staff training [median of USD 3,316 in 2012 and USD 2,913 in 2016], site management [median of USD 3,787 in 2012 and USD 6,175 in 2016]).

**Table 1:** Retrospectively estimated working times and costs to plan and prepare RCTs.

	2012					2016				
		Costs (in USD)		Working time (in days)			Costs (in USD)		Working time (in days)	
	Sample size	Median (mean)	IQR (min-max)	Median (mean)	IQR (min-max)	Sample size	Median (mean)	IQR (min-max)	Median (mean)	IQR (min-max)
Total	18	72,431 (77,182)	59,435–87,638 (13,977–180,809)	113.1 (121.7)	51.3–189.8 (20.8–241.8)	35	72,631 (111,602)	42,611–169,540 (11,907–366,653)	132.6 (192.4)	79.3–239.2 (37.7–971.1)
Single centre	9	74,289 (78,560)	59,435–87,638 (13,977–180,809)	143.0 (132.6)	82.6–189.8 (24.1–211.9)	26	62,253 (91,392)	36,811–151,931 (11,907–260,458)	106.0 (156.8)	58.5–217.6 (37.7–458.9)
Multicentre	9	67,704 (76,709)	50,140–101,048 (13,977–180,809)	106.6 (110.7)	51.3–126.1 (20.8–241.8)	9	140,483 (169,983)	87,543–221,030 (62,077–366,653)	193.7 (295.5)	132.6–288.6 (92.3–971.1)
Non-industry	16	67,704 (76,709)	50,140–101,048 (13,977–180,809)	107.9 (112.3)	45.2–164.5 (20.8–241.8)	31	72,631 (117,727)	44,058–176,534 (11,907–366,653)	135.2 (202.7)	80.6–288.6 (39.7–971.1)
Industry	2	80,963 (80,963)	74,289–87,638 (74,289–87,638)	196.3 (196.3)	189.8–202.8 (189.8–202.8)	4	57,581 (64,130)	36,624–91,637 (31,665–109,692)	72.5 (87.9)	39.4–136.5 (37.7–169.0)
No CTU involvement	11	79,731 (88,262)	59,435–128,845 (13,977–180,809)	143.0 (143.4)	68.9–211.9 (24.1–241.8)	20	73,106 (101,090)	44,192–140,917 (28,609–260,458)	133.9 (177.8)	89.4–228.8 (41.0–500.5)
With CTU involvement	7	60,388 (59,770)	40,844–73,609 (37,953–74,289)	82.6 (87.5)	39.0–109.2 (20.8–202.8)	8	52,645 (114,124)	26,931–187,638 (11,907–366,653)	76.1 (235.0)	49.4–310.3 (37.7–971.1)
Risk category A	NA	-	-	-	-	29	73,582 (111,801)	44,058–169,540 (11,907–295,515)	135.2 (180.3)	86.5–239.2 (39.7–500.5)
Risk category B	NA	-	-	-	-	3	70,914 (153,255)	22,198–366,653 (22,198–366,653)	79.3 (368.8)	55.9–971.1 (55.9–971.1)
Risk category C	NA			-	-	3	62,679 (68,012)	31,665–109,692 (31,665–109,692)	93.6 (100.1)	37.7–169.0 (37.7–169.0)

Abbreviations: CTU = clinical trial unit; IQR = interquartile range; min = minimum; max = maximum; NA = not applicable.

**Table 2:** Sensitivity analysis results regarding retrospectively estimated working times and costs to plan and prepare RCTs.

	2012			2016		
	n	Median (mean)	IQR (min-max)	n	Median (mean)	IQR (min-max)
**Total working time (in days)**						
Main analysis	18	113.1 (121.7)	51.3–189.8 (20.8–241.8)	35	132.6 (189.6)	79.3–239.2 (37.7–971.1)
Scenario 1	18	87.0 (93.6)	39.5–146.0 (16.0–186.0)	35	102.0 (145.8)	61.0–184.0 (29.0–747.0)
Scenario 2	18	87.0 (93.6)	39.5–146.0 (16.0–186.0)	35	102.0 (145.8)	61.0–184.0 (29.0–747.0)
Scenario 3	12	134.6 (135.3)	75.7–196.3 (24.1–232.7)	14	93.0 (122.6)	57.4–135.2 (37.7–500.5)
**Total costs (in USD)**						
Main analysis	18	72,431 (77,182)	59,435–87,638 (13,977–180,809)	35	72,631 (111,602)	42,611–169,540 (11,907–366,653)
Scenario 1	18	57,458 (61,530)	45,919–68,000 (10,869–147,064)	35	58,246 (87,980)	34,078–130,651 (9,253–283,881)
Scenario 2	18	50,107 (52,834)	38,410–57,092 (9,142–128,318)	35	49,726 (74,856)	28,517–109,046 (7,779–257,681)
Scenario 3	12	75,635 (83,213)	59,744–108,241 (13,977–180,809)	14	55,596 (70,548)	31,665–87,543 (22,198–221,030)

Main analysis: +30% non-pre-specified time, +20% overhead on salaries, complete datasets but including imputed salaries.Scenario 1: +0% non-pre-specified time, +20% overhead on salaries, complete datasets but including imputed salaries.Scenario 2: +0% non-pre-specified time, +0% overhead on salaries, complete datasets but including imputed salaries. Scenario 3: +30% non-pre-specified time, +20% overhead on salaries, complete datasets without imputed salaries. Abbreviations: IQR = inter quartile range; min = minimum; max = maximum.

**Table 3:** Item-specific costs to prepare RCTs.

	2012			2016		
Item-specific costs (in USD)	n	Median (mean)	IQR (min-max)	n	Median (mean)	IQR (min-max)
Protocol/forms	18	34,774 (41,457)	24,610–64,078 (6,941–76,771)	43	39,503 (61,188)	20,560–88,268 (4,116–312,007)
Budget	16	1,462 (1,859)	677–2,025 (255–5,954)	35	1,968 (4,005)	1,018–5,0489 (318–20,375)
Communication	18	7,322 (12,122)	4,621–15,126 (690–33,707)	40	6,506 (12,471)	3,048–15,253 (694–65,851)
Staff training	15	3,316 (5,103)	1,139–8,288 (725–15,113)	34	2,913 (6,088)	1,382–4,555 (136–45,850)
Site management	14	3,787 (5,441)	1,782–5,525 (255–16,621)	35	6,175 (7,993)	3,514–10,805 (442–39,932)
Database	16	2,124 (4,589)	1,450–4,558 (1,005–17,958)	35	2,487 (4,843)	1,105–4,614 (273–33,153)
Biobank setup	1	518 (518)	- (-)	7	1,658 (1,633)	884–2,038 (580–3,209)
Fees	19	1,782 (2,910)	815–4,073 (509–12,831)	44	1,527 (3,124)	815–3,055 (285–19,348)
Insurance costs	4	7,452 (11,124)	3,883–18,366 (3,564–26,030)	3*	611 (883)	509–1,527 (509–1,527)
Support	7	2,037 (9,726)	815–14,664 (163–40,732)	10	3,310 (6,176)	509–6,110 (204–25,458)
Travel expenses	8	2,546 (2,712)	650–4,400 (200–6,000)	12	2,037 (2,415)	754–4,073 (20–6,110)
Other fixed costs**	2	6,293 (6,293)	3,422–9,165 (3,422–9,165)	4	15,274 (53,589)	5,346–101,831 (509–183,296)

*These three RCTs were classified as risk category A.

**Other fixed costs referred to (if specified) piloting or amendment of the study protocol; administrative tasks; preparation of patient enrolment, intervention or biological markers.

Abbreviations: IQR = inter quartile range; min = minimum; max = maximum.

We collected approval time data from Swiss RECs for a total of 183 and 217 RCTs in 2012 and 2016, respectively. Approximately half of the included RCTs were industry-sponsored and more than two thirds were multicentre (Table 4). The vast majority of approved multicentre RCTs were international; only 23 (16%) in 2012 and 33 (22%) in 2016 focused exclusively on Switzerland. In 2016 there were 99 (45.6%) risk category A, 50 (23.0%) risk category B, and 68 (31.3%) risk category C RCTs.

**Table 4:** Time (in days) from submission of study documents of a planned randomized clinical trial until research ethics committee approval in 2012 and 2016.

		2012			2016	
	Sample size (%)	Median (mean)	IQR (min-max)	Sample size (%)	Median (mean)	IQR (min-max)
Total	183 (100.0)	72 (85.0)	41–107 (1–329)	217 (100.0)	109 (128.6)	79–154 (20–467)
Single centre	40 (21.9)	87 (91.4)	51–113.5 (14–329)	68 (31.3)	97 (121.0)	71.2–141 (29–393)
Multicentre	143 (78.1)	70 (83.2)	38–105 (1–320)	149 (68.7)	114 (132.1)	85–155 (20–467)
National	23 (16.1)*	76 (96.3)	45–115 (1–320)	33 (22.1)*	103 (123.2)	75–135 (20–467)
International	120 (83.9)*	70 (80.7)	37.5–104 (5–301)	116 (77.9)*	120 (134.6)	87–161 (21–322)
Non-industry	89 (48.6)	67 (85.5)	35–107 (1–329)	129 (59.4)	99 (124.7)	74–145 (20–467)
Industry	94 (51.4)	81.5 (84.6)	46–106 (5–292)	88 (40.6)	117.5 (134.4)	87–162.5 (21–322)
Risk category A	-	-	-	99 (45.6)	97 (114.6)	70–136.5 (20–467)
Risk category B	-	-	-	50 (32.0)	110 (138.0)	85.2–158 (38–393)
Risk category C	-	-	-	68 (31.3)	134.5 (142.1)	88–175 (50–322)

Abbreviations: IQR = interquartile range; min = minimum; max = maximum.

* Percentage refers to total of multicentre trials (n = 143).

The median time from REC submission to approval was 72 (IQR: 41–107) days in 2012 and 109 (IQR: 79–154) days in 2016 (Table 4). The duration from submission until first response from the REC was 25 (IQR: 18–42) days in 2012 (information missing for 20 studies) and 36 (IQR: 27–49) days in 2016 (S1 Table). The duration from first response from the REC until final approval was 42 (IQR: 16–76) days in 2012 (information missing for 20 studies) and 63 (IQR: 41–106) days in 2016 (S2 Table). The data received from RECs did not allow us to subtract times which sponsors required to implement requested adaptions. For the subgroup of single-centre RCTs, the median time from submission to REC approval was 87 (IQR: 51–114) days in 2012 and 97 (IQR: 71–141) days in 2016 (two-sided Wilcoxon rank-sum test for unpaired samples: p = 0.043).

For 2012 we received data for 213 clinical trials and the median duration from receiving a dossier until the final decision by Swissmedic (without counting the time which the sponsor used to provide requested amendments) was 25 (IQR: 18–33; Table 5) days. In 2016 a total of 179 clinical trials were evaluated, with a median time to Swissmedic decision of 36 (IQR: 33–38) days. Of note, for a few clinical trials submitted to Swissmedic in 2012 the date when amendments were requested from the sponsor was not noted, hence the durations for 2012 might be overestimated. The total duration from receiving a dossier until the final decision (not subtracting the days a sponsor used to implement requested amendments) was, in median, 27 (IQR: 19–51) days in 2012 and 49 (IQR: 36–67) days in 2016. Combined Swissmedic and RECs approval times could not be assessed as explained above.

**Table 5:** Swissmedic approval times by year and sponsor for clinical trials (not exclusively randomized clinical trials).

		2012			2016		
	Sponsor	Sample size	Median (mean)	IQR	Sample size	Median (mean)	IQR
Approval time (in days) Swissmedic (excluding time sponsor used to implement requested amendments)	All	213	25.0 (27.2)	17.8–33.0	179	36.0 (37.9)	33.0–38.0
	Industry	143	22.5 (24.3)	16.0–33.0	120	36.0 (38.0)	34.0–37.0
	IIT	52	28.0 (34.5)	20.5–43.0	42	36.0 (36.0)	32.0–38.3
	Collaborative study groups	18	27.0 (27.3)	17.8–36.3	17	37.0 (41.8)	33.0–53.0
Approval time (in days) Swissmedic (including time sponsor used to implement requested amendments)	All	213	27.0 (48.0)	19.0–50.5	179	49.0 (55.1)	36.0–67.0
	Industry	143	23.0 (34.1)	16.0–50.0	120	42.0 (50.4)	36.0–62.0
	IIT	52	44.5 (85.4)	25.0–134.0	42	56.5 (62.0)	40.5–79.0
	Collaborative study groups	18	28.5 (56.9)	21.0–86.5	17	72.0 (70.9)	48.0–88.0

Abbreviations: IQR = interquartile range; IIT = Investigator initiated trial.

Interpharma provided data on approval times in an aggregated form for a total of 71 clinical trials (randomized and non-randomized) in 2012 and 2013 (separate data for 2012 alone were not available) conducted at more than 200 study sites by 14 pharmaceutical companies. The median approval time (i.e. from first submission until final approval) at a lead REC was 64 (mean: 73; IQR: not available for Interpharma data) days and the median approval time at a non-lead REC was 31 (mean 23) days. The median duration to obtain Swissmedic approval in 2012 and 2013 was 22 (mean 25) days. For 2016, Interpharma provided aggregated data on approval times from 64 clinical trials conducted at approximately 180 study sites by 10 different pharmaceutical companies. The median approval time in 2016 was 95 (mean 110) days for a lead REC, 100 (mean 98) days for a non-lead REC, and 76 (mean 76) days for approval from Swissmedic.

## Discussion

### Main findings

Reports of rapidly rising clinical trial costs worry researchers all over the globe and Dr. Claiborne, Director of the University of California, for instance, stated that “reducing the costs of trials is absolutely crucial for the public good” [19]. However, to be able to identify suitable lever-points to reduce clinical trial costs it is essential that cost data are available. This study underlined that collecting cost data (i.e. data on resource use and preparation costs) for RCTs in Switzerland is difficult since these data are usually not documented in a systematic way. Based on a small sample with complete data, the median estimated costs to plan and prepare an RCT were very similar in 2012 and in 2016 (median USD 72,500), with similar ranges of costs also at the level of individual items. These costs are substantial, especially when considering that large parts of the preparation and planning of academic RCTs are typically done before the funding is actually secured. Specific cost items may finally not be reimbursed, as the work was done before applying for a research grant (e.g. systematic literature search, sample size calculation). When analysing approval times separately for the two authorities (i.e. RECs and Swissmedic), approval times appeared to be both longer in 2016 than in 2012. Data from industry-sponsored trials provided by Interpharma suggested an even larger difference in approval times between 2012/2013 and 2016, which may be due to biased sample selection. We were not able to link data from Swiss RECs (or BASEC) and Swissmedic for individual RCTs. Therefore, combined approval times for RCTs requiring REC and Swissmedic approval could not be assessed.

### Strengths and limitations

Our study has the following strengths: It is the first study to assess working efforts and costs to plan and conduct RCTs following an empirical and quantitative approach [9]. Furthermore, we transparently list different items together with information about median cost which might be helpful for other research in the planning and preparation phase of their RCTs. We evaluate a potential impact of the Swiss LHR on RCT-associated resource use and costs, using a before and after study design. Although we received only limited data, consistency in costs at item level (comparing 2012 and 2016) may indicate that selection effects did not lead to major distortions in this analysis. We also made the most comprehensive effort to date to compare approval times from competent authorities. We received approval time data from three different sources (i.e. RECs/BASEC, Swissmedic, and Interpharma), all indicating that separate approval times for RECs and Swissmedic were longer in 2016 compared to 2012. While approval time data collected by Interpharma probably suffer from selection effects (voluntary data entry by pharmaceutical companies), the data from RECs/BASEC and Swissmedic are complete census data for 2012 and 2016 and, therefore, highly representative for Switzerland.

In terms of limitations, only a relative small sample of cost data could be retrieved. Especially with respect to industry-sponsored RCTs and RCTs approved in 2012, the achieved sample size was non-satisfactory. Thus, a distortion of the observed lack of a difference in trial preparation costs between 2012 and 2016 due to recall problems and selection effects cannot be ruled out. A second limitation is that a large proportion of RCTs approved in 2016 were category A studies (99 of 217 [45.6%]). For these studies, Swissmedic approval is no longer required under the new LHR. In 2012, this categorisation did not yet exist and all RCTs had to undergo Swissmedic approval. Hence, the Swissmedic approval times for 2012 and 2016 cannot necessarily be assumed to apply to the same 'population' of RCTs, limiting comparability. The higher risk RCTs that still require Swissmedic approval may need more time than was needed earlier, on average. Third, it would have been important to estimate combined approval times (from first submission to either first REC or Swissmedic until last approval by REC or Swissmedic), given that in the trials category B and C studies still requiring Swissmedic approval under the new LHR, submissions can be done in parallel while they had to be sequential under the old legislation. As REC and Swissmedic data did not share a common trial identification number that could have been used to match information between the sources, we could only study REC and Swissmedic approval times separately [10]. Fourth, approval times were retrieved differently in 2012 (directly from RECs) compared to 2016 (from BASEC). Therefore, the 2012 sample included approval times for multicentre RCTs from lead RECs as well as from non-lead RECs while the 2016 sample only included approval times from lead RECs. We were not able to consistently distinguish lead RECs and non-lead RECs in 2012, because the lead REC process was only partially implemented at the time. Self-reported data from pharmaceutical companies collected by Interpharma, however, distinguished lead and non-lead RECs already for 2012 and 2013. Fifth, we did not consider drug development phases in our analysis since such information was not available and since the concept would not be directly applicable to non-drug trials. The REC data did not allow us to study approval times excluding investigator response times, other than in the case of Swissmedic. Here, the differences between 2012 and 2016 were similar irrespective of whether investigator response times were included or excluded. Sixth, the data from Swissmedic and Interpharma also included non-randomized trials; Swissmedic stated that the role of non-randomized trials was marginal. Seventh, changes in approval times may also have occurred due to general differences in approved studies (e.g. increasing numbers of dossiers may be submitted by non-Swiss companies which are not familiar with the Swiss legislation).

### Comparison with similar studies

We are not aware of any other studies that investigated preparation costs for RCTs to which we could compare our results to [9].

In terms of investigations of approval times, Heerspink and colleagues [20] conducted a study in which they assessed if the European Union Clinical Trials Directive in 2001 facilitated clinical drug research. To approve EU sites by authorities which adhered to the directive took significantly longer (median 75 days) when compared with EU sites in which local legislations applied (median 59 days). The authors concluded that the new directive “appears not to shorten the duration of regulatory procedures within Europe”. However, it remained unclear how the data were collected and how large the sample size was [20]. Another study assessed approval times from 16 tuberculosis trials in South Africa [21]. They found median approval times of 122 (IQR: 112–168) days for the Medicines Control Council and 60 (IQR: 33–81) days for human research ethics committees. They concluded that the drug development phase represented by a trial or support from a CRO had no effect on the approval time [21]. Furthermore, we identified two studies which both assessed REC approval times for a large multicentre RCT [22, 23]. While Kenyon et al. described approval times only qualitatively for different countries for the STITCH II trial (Surgical Trial in Lobar Intracerebral Haemorrhage) [22], Lutz and colleagues found a median RECs approval time of 75 (IQR: 42–150) days [23]. These published approval times are all in a similar range as found in our study, suggesting similar processes and challenges internationally.

## Conclusions

We found that the preparation costs of RCTs in Switzerland were approximately USD 72,000 in 2012 and in 2016. We believe that these costs are high for academic researchers when considering that several working steps may have to be taken before funding is awarded. Interestingly, some items which may often be forgotten in cost calculations (such as communication, staff training, and site management) cost several thousand USD. Approval times for single centre RCTs with Swiss RECs appeared to be longer in 2016 than in 2012. In addition, our empirical study revealed the following (1) the collection of RCT preparation cost data via a large investigator survey was unsatisfactory with results based on a small sample and, therefore, prone to selection, recall, and chance effects. We had the impression, that costs are often not documented in investigator-initiated RCTs, hence it required large efforts to retrospectively estimate the costs. Simple and freely available tools to plan and track trial costs might help to record cost data. From several industry initiated RCTs we received the answer that they cannot provide us with these data due to confidentiality issues. (2) A simultaneous analysis of the true combined REC and Swissmedic approval times for individual RCTs was not feasible, because data from Swiss RECs (or BASEC) and those provided by Swissmedic could not be linked at the RCT level. For effective and valid research on RCT approval times direct collaboration between stakeholders with data linkage is necessary. (3) Available Interpharma data suggested more extreme increases in approval times from 2012/2013 to 2016, probably due to selection effects. In view of the pending revision of the LHR, adaptations should be considered so that all data necessary to empirically evaluate the LHR are provided to the authority charged with the enforcement of the HRA (i.e. the Federal Office of Public Health), with appropriate measures for data protection. Without valid empirical data, consequences and impact of the LHR on the research enterprise in Switzerland cannot be accurately assessed.

## Supporting information

S1 File STROBE checklist. (DOCX)

S2 File Developed tool to collect resource use and costs from individual randomized controlled trials. (XLSX)

S1 Table Time (in days) from submission to research ethic committee until first response in 2012 and 2016. * Proportion based on multicentre randomised controlled trials. ^a^ Number of missing trials: 20 (10.9%). Abbreviations: IQR = inter quartile range; max = maximum; min = minimum; REC = research ethics committee.
(DOCX)

S2 Table Time (in days) from first response from research ethic committee until approval in 2012 and 2016. *Proportion based on multicentre randomised controlled trials. ^a^ Number of missing trials: 20 (10.9%). Abbreviations: IQR = inter quartile range; max = maximum; min = minimum; REC = research ethics committee.
(DOCX)
