# Supplementary material for: Resource use, costs, and approval times for planning and preparing a randomized clinical trial before and after the implementation of the new Swiss human research legislation
Source: PLoS One. 2019 Jan 11;14(1):e0210669. doi: 10.1371/journal.pone.0210669 (PMC6329511; doi:10.1371/journal.pone.0210669)
Supplement: S1 Table — * Proportion based on multicentre randomised controlled trials. a Number of missing trials: 20 (10.9%). Abbreviations: IQR = inter quartile range; max = maximum; min = minimum; REC = research ethics committee. (DOCX) [file pone.0210669.s003.docx]

**Table S1**: Time (in days) from submission to research ethic committee until first response in 2012 and 2016.

|  |  | **2012** |  |  | **2016** |  |
| --- | --- | --- | --- | --- | --- | --- |
|  | **n (%)**^a^ | **Median**  **(mean)** | **IQR**  **(min-max)** | **n (%)** | **Median**  **(mean)** | **IQR**  **(min-max)** |
| Total | 163 (100.0) | 25  (33.3) | 17.5-41.5  (1-165) | 217 (100.0) | 36  (45.2) | 27-49  (10-247) |
| Single centre | 35 (21.5) | 30  (41.3) | 21-51.5  (4-157) | 68 (31.3) | 34  (47.5) | 27-46.5  (16-240) |
| Multicentre | 128 (78.5) | 24  (31.1) | 17-37  (1-165) | 149 (68.7) | 38  (44.2) | 26-49  (10-274) |
| National | 20 (15.6)* | 21.5  (32.0) | 13.8-37  (1-165) | 33 (22.1)* | 29  (38.8) | 21-41  (10-193) |
| International | 108 (84.4)* | 24  (31.0) | 17-37  (1-131) | 116 (77.9)* | 41.5  (45.8) | 28-50  (15-274) |
| Non-industry | 78 (47.9) | 25  (34.9) | 17-40  (1-165) | 129 (59.4) | 34  (48.2) | 26-50  (10-274) |
| Industry | 85 (52.1) | 25  (31.8) | 18-42  (2-120) | 88 (40.6) | 40  (40.9) | 27-48  (15-99) |
| Risk category A | - | - | - | 99 (45.0) | 34  (50.8) | 22.5-51.5  (10-274) |
| Risk category B | - | - | - | 50 (32.0) | 35.5  (37.7) | 27-44  (14-76) |
| Risk category C | - | - | - | 68 (31.3) | 41  (42.8) | 29.8-49  (15-125) |

* Proportion based on multicentre randomised controlled trials.

^a^ Number of missing trials: 20 (10.9%).

Abbreviations: IQR=inter quartile range; max=maximum; min=minimum; REC=research ethics committee.
