# Supplementary material for: Resource use, costs, and approval times for planning and preparing a randomized clinical trial before and after the implementation of the new Swiss human research legislation
Source: PLoS One. 2019 Jan 11;14(1):e0210669. doi: 10.1371/journal.pone.0210669 (PMC6329511; doi:10.1371/journal.pone.0210669)
Supplement: S2 Table — *Proportion based on multicentre randomised controlled trials. a Number of missing trials: 20 (10.9%). Abbreviations: IQR = inter quartile range; max = maximum; min = minimum; REC = research ethics committee. (DOCX) [file pone.0210669.s004.docx]

Table S2: Time (in days) from first response from research ethic committee until approval in 2012 and 2016.

|  |  | **2012** |  |  | **2016** |  |
| --- | --- | --- | --- | --- | --- | --- |
|  | **n (%)^a^** | **Median**  **(mean)** | **IQR**  **(min-max)** | **n (%)** | **Median**  **(mean)** | **IQR**  **(min-max)** |
| Total | 163 (100.0) | 42  (54.8) | 15.5-75.5  (0-281) | 217 (100.0) | 63  (83.4) | 41-106  (0-357) |
| Single centre | 35 (21.5) | 47  (55.0) | 22.5-68  (0-281) | 68 (31.3) | 47.5  (73.5) | 29-103  (0-357) |
| Multicentre | 128 (78.5) | 41.5  (54.7) | 12-78  (0-271) | 149 (68.7) | 72  (87.8) | 44-110  (0-348) |
| National | 20 (15.6)* | 53.5  (66.8) | 30-87  (0-241) | 33 (22.1)* | 70  (84.4) | 35-99  (0-348) |
| International | 108 (84.4)* | 39.5  (52.5) | 5-70.5  (0-271) | 116 (77.9)* | 76.5  (88.8) | 49-114  (0-268) |
| Non-industry | 78 (47.9) | 37  (53.0) | 14-65.2  (0-281) | 129 (59.4) | 53  (76.5) | 33-102  (0-357) |
| Industry | 85 (52.1) | 47  (56.4) | 23-84  (0-271) | 88 (40.6) | 79.5  (93.5) | 50-119  (0-268) |
| Risk category A | - | - | - | 99 (45.0) | 48  (63.8) | 30-84  (0-274) |
| Risk category B | - | - | - | 50 (32.0) | 75  (100.4) | 46.5-111  (9-357) |
| Risk category C | - | - | - | 68 (31.3) | 90.5  (99.4) | 49-124  (12-268) |

*Proportion based on multicentre randomised controlled trials.

^a^ Number of missing trials: 20 (10.9%).

Abbreviations: IQR=inter quartile range; max=maximum; min=minimum; REC=research ethics committee.
